# Solidarity, not charity: Learning the lessons of the COVID‐19 pandemic to reconceptualise the radicality of mutual aid

**DOI:** 10.1111/tran.12553

**Published:** 2022-07-01

**Authors:** Oli Mould, Jennifer Cole, Adam Badger, Philip Brown

**Affiliations:** ^1^ Department of Geography Royal Holloway University of London London UK; ^2^ Department of Behavioural and Social Sciences University of Huddersfield Huddersfield UK

## Abstract

The COVID‐19 pandemic has significantly ruptured our global society. We have seen health care systems, governments and commerce buckle under the strain of disease, lockdowns and unrest, but the rupture has also created space for radical (and anarchist) politics of mutual aid, as societal organising principles, to move into a more prominent position (and offers potential for this shift to remain after the crisis has subsided). However, in the short time since mutual aid has been thrust into the limelight, we have seen a multiplicity and spectrum of geographies, applications and approaches. Indeed, we have also seen its appropriation by government(s) that takes advantage of mutual aid's rallying cry of “solidarity not charity”; absolving the state's responsibilities to sufficiently fund social welfare when good neighbours will do it for free. In this paper we map out how mutual aid has been enacted during the COVID‐19 pandemic by charity, contributory and radical groups to address specific and novel forms of vulnerabilities, and the opportunities and challenges this offers for the future. In particular we highlight potential tensions between the enacting of mutual aid practices and the political activism (or not) of the mutual aid actors. Our contribution is to reconceptualise mutual aid to (i) show where the real “mutualism” of mutual aid is, and (ii) create a better understanding of how mutual aid can be mobilised in future emergencies which will inevitably arise in the current climate emergency.

## Abstract 2

This paper maps out how mutual aid has been enacted during the COVID‐19 pandemic by charity, contributory and radical groups; but brings this together with a specific understanding of vulnerability as a site of possibility rather than passivity to forward a reconceptualisation of mutual aid that is based far more on solidarity, than charity.

## 1 INTRODUCTION

As the majority of the world brought in the new decade with fireworks and celebrations on midnight 1 January 2020, a new and deadly virus was working its way from Wuhan, China, into virtually every country of the globe. Within a matter of months, the world was experiencing its most acute pandemic since the Spanish Flu of 1918–19, and numerous countries all over the world experienced multiple lockdowns. At the time of writing, the coronavirus pandemic is still ongoing, with the many variants of the SARS‐CoV‐2 virus embedding themselves into the long list of viral diseases that circulate in the human population. In addition, the social and geographical ramifications of the pandemic continue to reverberate around the countries of the world and their political regimes, and will do so for some foreseeable time (Cole & Dodds, 2021; Ho & Maddrell, 2021; Rose‐Redwood et al., 2020).

This is because the response, at all geographic scales and institutional levels, to the COVID‐19 pandemic has been instructive, not only for future potential public health emergencies but also for how we respond to similar transformative global events such as the climate catastrophe. Moreover, how communities respond to help each other in the wake of more devastating extreme weather events (such as the wildfires, storms and flooding witnessed in recent years) will require a major recalibration, particularly in relation to *institutionalised* responses; i.e., those from national charities, governments, corporations and other “top down” approaches that have the ability to affect the organisation of the local community.

The responses to the social ills (food poverty, loneliness, etc.) of the pandemic were scored along geographical lines tracing the different levels of *vulnerability* that exist, with traditionally more “resilient” (in the “top‐down” institutionalised reading of the term as critiqued by MacKinnon & Derickson, 2013) and hence less vulnerable areas generally being able to mobilise resources more quickly and more efficiently (e.g., the south‐east of England in comparison to the north‐west). As such, the mobilisation of responses to these differences in geographical resilience has likewise been varied, because how “vulnerable” people were (and still are) in these differing areas has a myriad of social, political, economic and, critically, geographical characteristics that the pandemic has drastically exposed and shifted.

As Solnit (2009) has identified, what happens in response to a disaster is often the “triumph” of collective civil action in the wake of real and perceived failures of institutional authority. But such responses are not evenly distributed across space when the resources available to some communities were already diminished or absent via the stripping away of local assets and resourcefulness (e.g., because of previous policies such as austerity in the UK; see Scrambler, 2020).

It is our contention that the time is ripe for a reconceptualisation of mutual aid. It is an important task to undertake at this particular juncture because the benefits of mutual aid – specifically its organising anarchist and anti‐capitalist geographies (Springer, 2014) – will not only help people who suffer because of climate catastrophe, but it may provide a platform to tackle the root causes of climate change itself: an over‐reliance on capitalist narratives of economic growth, consumerism and unsustainable living practices (Henderson, 2021; Klein, 2015). For geographers working at the coalface of this area, from anti‐capitalist, anarchist and activist/action researchers in marginalised communities to policy analysts (or simply those responding to the immediate needs of the communities around them), understanding how mutual aid, a concept which is widely used in the discipline, has shifted during the planetary “COVID event” (Mould, 2021) is vital.

This paper argues, then, that to emphasise the mutualism of mutual aid, this reconceptualisation needs to happen alongside and in conjunction with a (re)emphasis on the rather nebulous notion of *vulnerability*. Within geographical scholarship on mutual aid at present, vulnerability is either not considered as a structural factor of its ontology or, if it is, it is often a singular idea of being “in need” (e.g. Springer, 2020). Hence a more radical view of vulnerability – one that sees it as a site of possibility and connection rather than a state of passivity (Butler et al., 2016) – brings a new more constitutive concept of mutual aid into focus. This will resonate with anarchist geographies most closely (Ferretti, 2017; Ince, 2019; Pickerill, 2017; Springer et al., 2012), but also with the existing richness of geographical scholarship that uses the practice of mutual aid for detailing contemporary radical concepts such as social justice (e.g., Travlou, 2021; Williams & May, 2022), anti‐capitalism and climate action (Nelson, 2020; Pickerill, 2021), post‐statist geographies (Ince & Barrera de la Torre, 2016), anti‐racism (Elliott‐Cooper, 2021; Liebman et al., 2020) and urban commoning (Ruiz Cayuela, 2021; Tsavdaroglou & Kaika, 2022), among others.

To do this, the paper first gives a brief background to the anarchist geographies of mutual aid and how it has been enacted during the COVID‐19 pandemic. In so doing, the paper shows how it can be reconceptualised and where it might find itself in the post‐pandemic world, institutionally or otherwise. The paper considers whether the creeping co‐option of mutual aid by state‐level institutions such as national governments, NGOs and charities will dilute its radical anti‐capitalist policies, and thus deflate its political praxis by allowing it to be absorbed into the capitalist and institutionalised readings of “resilience” more broadly (MacKinnon & Derickson, 2013). Or, more positively, whether it is a welcome development that will position its radical tendencies more strongly in the mainstream political ecology of the post‐pandemic world and reconceptualise our understandings of how vulnerability is distributed spatially. This reconceptualisation of mutual aid will be presented along the lines of a three‐part model of *charity*, *contributory* and *activist* (adapted from, but then expanding initial groundwork by, Gulick et al., 2020).

Crucially, though, the paper extends geographical understanding of mutual aid further by combining it with specific and radical conceptualisations of vulnerability. Who is vulnerable and in need of “care” and “support” (and the role *mutual* aid plays in this) changes radically in a crisis, and harshly exposes geographical differences, who is available and able to provide it (i.e., furloughed workers, volunteers, temporarily closed community and faith‐based centres, etc.). The various governmental policies of lockdowns, furlough, welfare changes and health care provision from around the world have meant that some people who were “normally” not subject to socio‐political shocks suddenly found themselves vulnerable. Terms such as “new poor” (World Bank, 2020) and “temporarily vulnerable” (Bergstrand et al., 2015) have been used to describe these groups, and this will be an important part of how mutual aid is conceptualised going forward, both in the minds of those who provided and those who received such aid. Hence, by combining radical versions of mutual aid *together with* vulnerability, we present a version of post‐COVID community care that is rooted in not just a provision of aid, but in praxis of contesting the system that created that need for aid in the first place.

## 2 THE ORIGINS AND RECONCEPTUALISATION OF MUTUAL AID

“Mutual aid” was first popularised by Russian evolutionary biologist‐cum‐anarchist philosopher Peter Kropotkin in his 1902 essay collection “Mutual aid: a factor of evolution” (Kropotkin, 1902). In this work, Kropotkin presents cooperation and reciprocity as fundamental organising principles of human society (Peet, 2008). Researching examples from the animal kingdom, pre‐capitalist societies in Europe and indigenous communities from around the world, Kropotkin was adamant that the “survival of the fittest” paradigm was not the only “perpetuation of life”; mutual aid and cooperation between species was just as, if not more important, for evolutionary paradigms. Within geography, anarchist thinkers (e.g., Springer, 2012, 2014, 2020), have fused Kropotkin's ideas – pregnant as they are with the emancipatory potentials of mutual aid – with existing critical geographies of territory (Ince, 2012), statehood (Barrera de la Torre, 2021), electoral geographies (Araujo et al., 2017), science and knowledge (Ferretti, 2017), Blackness (Heynen & Rhodes, 2012), feminism (Ferretti, 2016; Mott, 2018) and many others. Within all of these applications, mutual aid is broadly conceptualised, as it was for Kropotkin, as the democratic cooperation, not competition, between individuals that made life flourish.

But within contemporary capitalist life of the twenty‐first century, predicated as it is on a near‐ubiquitous neoliberal mindset of competition for scarce recourses, mutual aid has been (even more so) relegated to a mode of operation that is useful for micro‐scale community operations, such as squats (Zaman, 2020), eco‐communes (Pickerill, 2021) and urban neighbourhood communities (Williams & Windebank, 2000), but not a valid means of organising society on a broader scale (Firth, 2020; Spade, 2020; Springer, 2020). As such, mutual aid has been most often thought of as a tool of radical resistance and anti‐ or post‐capitalist praxis (Nelson, 2020), but an ineffective and utopian mode of organising society (as Harvey (2017) is at pains to point out in his trenchant response to Springer (2014)).

However, there is no doubt that mutual aid has come to prominence again during the COVID‐19 pandemic as lockdowns have restricted mobility, isolated and enclosed individuals and entire communities, forcing (or reigniting) local community bonds as people worked from home, were reduced to walks around their neighbourhoods and saw at close range the inequalities on their doorstep. Indeed, Spade (2020, p. 131) argues that “mutual aid strategies will be the most effective way to support vulnerable populations to survive, mobilize significant resistance, and build the infrastructure we need for the coming disasters.” The coronavirus pandemic, therefore, has seen a significant expansion in mutual aid networks precisely because the overarching capitalist system has proven to be largely incapable of providing for those with particular vulnerabilities, at least in the short term. Indeed, as Springer (2020, p. 113) noted, during the pandemic it is “reciprocity that is saving us from complete catastrophe”.

These mutual aid groups have expanded to help people in their hour of need, but so too has the definitional boundaries of the term. As the pandemic has progressed, more of these community‐led mutual aid networks have developed into, along with, despite of and in opposition to more institutional forms of charitable giving or governmental programmes of welfare. The term “mutual aid” therefore has been loosely applied to a myriad of different forms of organising, some of which align with the anarchist geographical traditions (outlined above), while others characterise its polar political opposites. For instance, in the United Kingdom, the Conservative MP Danny Kruger penned a government report that focused on “Levelling up” communities entitled “The new social covenant” (Kruger, 2020). In it, the term “mutual aid” appears 10 times, but in the context of volunteers, charities and those who gave up their time to help those in need: nowhere in it does the distinctly anarchist tendencies of mutual aid, and its distinct rejection of deeply capitalist notions of charity, appear. Whilst such myopia is to be expected in a report written by a Conservative politician, it does evidence a creeping appropriation of the term to account for activities that are not actually mutual at all.

The differing geographies of the socio‐political origins of mutual aid are stark when you consider how mutual aid is conceptualised in the United States, with its roots very much in Black communities who have organised among themselves in lieu of support from structurally racist state mechanisms (Aberg‐Riger, 2020; Heynen, 2009; Heynen & Rhodes, 2012). When covered as part of the response to the pandemic in the US press, mutual aid's legacy is celebrated as a contribution of Black culture to the current crisis. Indeed, one of the United States' earliest mutual aid organisations was the “Free African Society” in Philadelphia, which was instrumental in helping the city's population survive the Yellow Fever epidemic in 1793 (Aberg‐Riger, 2020). During this most recent pandemic, the US self‐proclaimed socialist senator Alexandria Ocasio‐Cortez produced a “mutual aid toolkit” to help affected communities. Within her material she referred directly to Kropotkin and repeatedly used the slogan “solidarity not charity”.

The distinct difference in the trans‐Atlantic geographies of how mutual aid has been applied by politicians and the communities “on the ground” is stark, and arguably forms part of a broader difference in conceptualisation of mutual aid praxis that is embedded in varying cultural histories and geographies as much as current over‐riding political ideologies (Beito, 2000). But given the prominence of the mobilisation of mutual aid *practice* during (and no doubt after) the COVID‐19 pandemic, focusing on the variations of how the mutual aid *conceptualisation* has developed allows for a more considered approach to understanding its role and political impact. In essence, mapping out how mutual aid as an idea has been mobilised by different groups (with very differing political motivations) provides a conceptual geographical toolkit with which individuals, neighbourhoods, community groups, activist networks and, yes, even national governments can identify – and perhaps then act upon – the objective differences between *mutual* aid and aid; between charity and solidarity. Hence, the next section will explore the nuances of how mutual aid has been conceptualised in past geographical histories, and how this has changed because of the COVID‐19 pandemic. In so doing, we present a lose triumvirate of mutual aid practices, gleaned from existing literatures (notably Gulick et al., 2020), groups, examples and current practices: *charity*, *contributory* and *radical*: it is to each we now turn.

### 2.1 Charity

In response to the coronavirus pandemic, the most visible “responders” to those in immediate need were individuals who saw or perceived those around them as vulnerable. Be they simply concerned neighbours, members of local volunteer groups, faith networks, schools, mental health services, food banks or soup kitchens, there were a plethora of responsible, caring and/or concerned people, sometimes already linked to quasi‐autonomous institutions, who were able to quickly mobilise people, finance and food so as to help those who were isolating, shielding or just generally in need of food and emotional support. Some organised through leafleting their local community, or on Facebook and WhatsApp. The modus operandi of these groups was first and foremost altruistic/philanthropic (Matthewman & Huppatz, 2020); to get food and support to those who cannot access it.

This kind of mobilisation from within affected communities is a long‐standing “model” that dates back to the “friendly societies” of Britain and the US in the nineteenth century, which were groups of largely working‐class men (with some women) who pooled financial resources to provide basic welfare services to all (Harris & Bridgen, 2012). Over time, particularly in Britain, these groups mutated into private enterprises (particularly insurance firms) that began to employ people to provide resources to those who needed them most. Today, we have what is called “conscience capitalism” (Farrell, 2015) that sees a working combination of for‐profit and not‐for‐profit enterprises, creating a whole new “market” consisting of social and environmental “externalities”. Under the rubric of so‐called “third way” neoliberalism (Brown, 2015; Peck, 2004), there exists now a harmony between marketisation and community action in which they become almost interchangeable; indeed social (and now environmental) responsibility undergirds and affirms marketised logics (Brown, 2015). Hence, charities in the twenty‐first century operate under strict neoliberal market logics, often competing for funding from national governments and philanthropic billionaires to provide aid to marginalised people (Scullion et al., 2015) and/or countries and/or regions (Mawdsley, 2012). And they do this while trying to keep their own wage budgets low, leading to the exclusion of anyone except the affluent (or generally time‐rich people) being able to “volunteer” with them. This process therefore “undermines its potential for democratic governance” (Farrell, 2015, p. 266) because it maintains existing capitalist hierarchies and keeps communities in a state of dependency on the aid provided rather than able to provide for themselves from within the community. Geography has a rich tradition of analysing how capitalist power appropriates and encloses “external” subjects for profitable gain at the corporate level (see Doshi & Ranganathan, 2019), and so applying this same critical lens to charities provides a much‐needed corrective to the neoliberalisation of charitable work at a variety of scales.

That is because within the COVID‐19 pandemic response, this same process of appropriation is playing out at the *local* scale (Chevée, 2021). As the various lockdowns across the world ruptured the smooth flow of capitalist goods and services, leaving many workers furloughed or redundant and children without access to the free school meals on which they were dependent for food or internet‐enabled devices on which they were dependent for education, the community groups stepped in and continued to provide these forms of welfare where corporations and government did not (at least in the short term). Within these cases, the term “mutual aid” was used rather liberally, largely because of the ways in which it mobilised individuals (rather than institutions) to provide the services. However, this operated as a form of “volunteerism” (Vrasti & Montsion, 2014), that is a *performance* of the idealised philanthropic neoliberal subject; one that un/wittingly undertakes a dutiful role to maintain the marketised logic of capitalist accumulation rather than undermine it.

Such charity‐like approaches to aid are by no means unwelcome: after all people are fed, receive their medications, the lonely and bereaved are comforted, and children are educated. But as a form of mutual aid, it lacks any of the *political* agency of the anarchist geographical literature, nor does it really aim to tackle the existing structures of injustice that caused the need for aid in the first place (Firth, 2020). It is mutual aid in name only.

Moreover, research (in the UK at least) has indicated that the demographics of the coronavirus volunteers have been majority white (90%), professional (76%), and female (84%) (O'Dwyer et al., 2022). Hence there is a gendered overture to this form of volunteerism which has roots in the histories of mutual aid. Harris and Bridgen (2012, p. 5) have noted how in the early twentieth century, the expansion of charitable companies provided “middle‐class women with opportunities for participation in public life that were otherwise denied them”. Given the structural gendered bias in domestic social work in the early twentieth century, those women who did less of this domestic care work than others (e.g., childless or retired women) often found they could perform such roles as employed charity workers. Such charity work was regarded as important for social ties: in essence, the role of women in this society was to maintain social “order” and to rear people for capitalist production. If this cannot be done at home for whatever reason, charities provided a way for this to be performed in the community. Hence, thinking about how the more recent mobilisation of aid during the pandemic has been scored along gendered (but also class, raced, disability and other intersectional lines) can help in adding further identity and embodied nuances to geographical research into mutual aid (cf. Brito & Jonas, 2021).

So, this charity model of mutual aid does little to dismantle the dominant mode of gendered, raced and unequal capitalist society; in fact it ossifies it. While clearly advantageous to the people they serve, it lacks the ethical mutualism (Mould, 2021) that has the potential to undermine capitalist logics that made people in need of service in the first place.

### 2.2 Contributory

In contrast, contributory groups go beyond a traditional charity model to try to actively champion the agency of the marginalised so as they can *contribute* to social life; they attempt to empower excluded and marginal groups to *contribute* to how society functions. Mutual aid in this conceptual framing empowers participation within the socio‐economic “system” as it currently operates.

We can again look to historical movement for examples: liberatory farming movements of Black Americans during the late nineteenth and early twentieth centuries saw many plantation slaves transition to being tenant farmers and eventually landowners working within farming co‐operatives (White, 2018). During the early years of the twentieth century, Fraternal Societies in the United States were the leading providers of life insurance and social welfare (Beito, 1990) while in the United Kingdom mutual building societies started in 1774 in Birmingham, enabling low‐paid workers to aspire to home ownership (Stefanelli, 2010).

Contributory forms of mutual aid (although by no means all) place a reliance on those who are marginalised (via their demography, ethnicity, health, etc.) to find the resources from within not only themselves but also their immediate community to aid in their own empowerment. In other words, while there is aid given, it often comes with resources and support that try to activate individual agency. This form of contributory mutual aid is often linked to a self‐help narrative, with groups formed around a particular issue (Borkman, 1999). Alcoholics Anonymous (AA) is perhaps one of the more famous examples of a contributory mutual aid group that uses the self‐help principles. AA identifies as an “international mutual aid fellowship” that is apolitical and “concerned solely with the personal recovery” (AA website, n.d., n.p.); but its mutual aid characteristics are obvious. AA “upended virtually every convention of organizational structure in a capitalist society” and among other anarchist principles, AA “eschews property ownership and rigorously refrains from soliciting support from sources other than its own members” (Gross, 2010, p. 2363). Starting out in 1935 as two men keeping each other sober in New York City and based on spiritual principles of “group conscience” (Kurtz, 2010), AA has maintained this structure largely unchanged for nearly a century and no doubt has saved countless lives in the process.

The apolitical nature of AA, though, means that while it has helped countless people, its energies are not focused on changing the system that can plunge people into alcoholism in the first place; it is part and parcel of the self‐help narrative that has become imbued into the well‐being economy (or what Purser, 2019 has rather acerbically called “McMindfulness”). Hence any anarchistic mutual aid characteristics that AA has, relates more to its organisational form rather than any potent political praxis. And as evidence of its co‐option into governmental mantras, Public Health England (PHE) makes an explicit link between mutual aid and the “fellowship” model of AA (alongside sister organisations such as Narcotics Anonymous and Cocaine Anonymous). In 2015, PHE published a report entitled “Improving mutual aid engagement” in which they argue that:

Mutual aid refers to the social, emotional and informational support provided by, and to, members of a group at every stage of their recovery from active alcohol and/or drug use and addiction. It is not a peer support network. It relies upon a structured programme that is *focused on recovery*. (Public Health England, 2015, p. 4, emphasis added).

The role, then, of AA and other “fellowship” models is about *recovery*; getting people back to a state of physical and mental health in order to contribute fully to – and be employed in – society again. The emphasis here, then, within these groups is on support for individuals to recover themselves; it is not primarily about a collective agency to change the structural and systemic conditions that saw them become alcoholics in the first instance.

Such a contributory framework of mutual aid has a great deal of commonality with the geographies of *resilience*, notably as part of a governmental and/or formalised institutional narrative of “resilience from above” (Vrasti & Michelsen, 2017; Weichselgartner & Kelman, 2015). Broadly, this is when people are expected to give up any desire for autonomy and security from the pressures of capitalist life, and instead to become a flexible and adaptable subject: someone (or a group) who is able to cope with exposure to a system that is by its very nature hazardous and exploitative (Reid, 2016). This has led to critiques of the very term “resilience” within geography and beyond (Cretney, 2014; MacLeavy et al., 2021), with a preference for discussing community “resourcefulness” (MacKinnon & Derickson, 2013) that recognises the agency needed to be present within a community to resist oppression and marginalisation. Using a “commons as praxis” mantra (which as anarchist geography will suggest is very much related to mutual aid; Springer, 2014), mutual aid can challenge individualism and embrace shared resources and co‐operative behaviours (Springer, 2014). This can lend some credence to the resilience literature, in which “resilience from below” (Solnit, 2009; Vrasti & Michelsen, 2017) is argued to have political motivations to change the conditions of survival. As such, the contributory model of mutual aid could find a greater deal of commonality with the geographies of resilience and the contestations therein. But to resist being co‐opted by a corporate language of “self‐help” or a governmental framework of “resilience from above”, it is perhaps pertinent to connect critical resilience geographies to the radical conceptualisation of mutual aid, to which we now turn.

### 2.3 Radical

Quoting Zižek (or Jameson, it has never quite been decided who), Fisher (2009, p. 1) prophesised that “it is easier to imagine the end of the world than the end of capitalism.” For charity and contributory groups, this maxim is undoubtedly true, as the inequality wrought by capitalism is positioned as a *fait accompli*, something to be addressed by remedial action in the community. Radical groups, however, seek to undermine the structural inequalities inherent to life under capitalism that create the conditions for inequality in the first place. Indeed, the slogan “solidarity not charity” (as stated above, proclaimed by Alexandria Ocasio‐Cortez in her COVID‐19 mutual aid documentation) stands in direct contradistinction to the philanthropic tenor that perpetuates charity models of mutual aid (Spade, 2020). So, whilst the contributory groups seek to build a “resilience” that make humans fit for life under capitalism, radicals help to turn this narrative on its head to challenge the underlying conditions that create, compose and perpetuate our deeply unjust social systems.

This ongoing commitment to radically re‐organising society to benefit the marginalised and oppressed, connects contemporary radical mutual aid efforts with a long tradition of the geographies of mutualisation and collectivisation. As Firth (2020, p. 58) argues, “mutual aid is part of a broader anarchist movement, which engages in more confrontational activities such as strikes and occupations as well as longer‐term co‐operative infrastructure and permaculture projects”, in order to defend a sphere of autonomous geographies from an appropriative capitalism (see also Pickerill & Chatterton, 2006). This lineage disconnects radicals from their charitable and contributory counterparts, instead bringing them into alignment with a long history of collectivised action against the forces of capitalist oppression (Springer et al., 2012). In doing so, they create spaces for those people dispossessed by mainstream social and political processes to empower themselves to change the structures that have disempowered them in the first place (Mould, 2021; Spade, 2020).

During the civil rights struggles of the late 1960s and 1970s, groups such as The Rainbow Coalition, founded by Fred Hampton and composed of Black Panthers (working‐class black activists), Young Patriots (working‐class white activists) and the Young Lords (working‐class LatinX activists), came together across intersectional differences to build spaces and capacities to campaign against the everyday fascisms of US society (Arguello, 2019). They operated in lieu of state architectures that left them behind, founding breakfast clubs and neighbourhood health clinics to pool resources and meet each others' immediate needs. In the UK, British‐Caribbean groups came together to form supplementary community schools to provide an education for their children who were deprived of it by a racist, exclusionary school system (Akala, 2019; Coard, 1971). Similarly, throughout the miners' strike of 1984–85, activists pooled and collectivised resources to fight for change and support striking miners with food parcels and funds to sustain them through their collective action (Bacharach et al., 2018). And more recently, the activist group in the US, “Food Not Bombs” (FNB) has been cooking vegan and vegetarian food in US cities' public squares and parks (often illegally and in the face of urban officials attempting to continually shut them down), in order to feed the homeless and urban poor (Parson, 2018). As such, the groups' radical mutualism is deliberately tied to their anti‐capitalist politics: indeed, as Heynen (2010, p. 1233) argued having conducted an in‐depth activist ethnography with the group, “unlike much charity, FNB works hard not to be complicit in the perpetuation of the capitalist states' biopolitics, but seeks to radically transform it.”

What these examples highlight is the recognition by radical mutual aid networks that people are unable to become fully active citizens in society if society does not enable them to meet their basic material needs for survival; and that once these are met, people are liberated to participate in civic life and form solidarities with one another to tackle the systems that marginalised them in the first instance. Therefore, rather than simply acting as disaster relief, radical forms of mutual aid seek to address the conditions that undermine our freedom and re‐enforce social inequalities; to eliminate the cause, rather than mitigate the symptoms of a crisis. For example, when discussing a Fred Hampton speech about the radicality of the Black Panthers' breakfast programme, Heynan (Heynen, 2009, p. 414) said Hampton “linked starvation of children across American inner cities to the destructive contradictions inherent in capitalism and the uneven development it produces”. The Black Panthers made an explicit link between the aid that was being provided and the injustices of the capitalist system that created a need for that aid.

One example of a contemporary mutual aid group operating in the radical tradition is Bristol‐based Acorn, “a mass membership organisation and network of low‐income people organising for a fairer deal for our communities” (Acorn, n.d.). They worked prior to the COVID‐19 pandemic to organise around issues of housing inequality in recognition of the fact that housing insecurity is often instrumental in bringing about increased precarity in people's lives (Brown et al., 2021; Waldron, 2021). They combine mass organising of a large network of members, with demonstrations and rent strikes to stop evictions, and to demand that safety standards are met and repairs are made. During the pandemic, Acorn members were able to deploy strategies that had already been honed through their activist struggles, harnessing capacity that had been built over the previous 6 years, since the group's formation, to provide immediate support to families in crisis. They are now working to highlight the ways in which the pandemic amplifies already latent crises in our communities, using this as a platform to build their movement, support the activist endeavours of other movements, and fight for long‐term social change.

Radical forms of mutual aid build on this tradition, and “is necessary to help fundamentally transform the conditions that created the crisis in the first place” and in relation to COVID‐19, this means accepting that “the emergency was here long before the virus arrived” (Gulick et al., 2020, n.p.). The result is therefore that mutual aid praxis can link with a multitude of geographical research trajectories that are focused around intersectional social justice issues (for a comprehensive contemporary overview of these, see Hopkins, 2021).

In a succinct summary of these three categories, charity groups tend to show no interest in changing the system (but help people in immediate need), contributory groups see the system as failing certain groups (and help bring those groups in line with others), while radical groups will see the system as fundamentally broken and call for complete change (and in doing so help bring about change that benefits the many, rather than the few). It can be further allegorised with the famous metaphor: charity groups will give someone a fish to eat, contributory groups will teach them how to fish, but radical groups will seize the means of fishing altogether.

This ideological triumvirate provides us with the “why/how” of a reconceptualisation of mutual aid and associated geographies, and whilst in itself marshals the ever‐expanding range of practices that are being swept up in the popularity of the term “mutual aid”, it requires a further dimension to aid in its *spatio‐political* application. In other words, the three different “models” outlined here are utilised differently across space depending on the already‐existing *vulnerability* of the community, and crucially, how this has increased (or not) during the pandemic. Hence, to understand the political lessons of how mutual aid has been mobilised during the COVID‐19 pandemic – and to reconceptualise it as a more useful political tool in the climate catastrophe‐ravaged future – it is equally as important to better our understanding of the “where/who” of mutual aid during the pandemic. Therefore, we need to meld this with the constantly shifting, geographically variable and rather transmutable conceptualisation of “vulnerability”.

## 3 VULNERABILITY

It has long been established across the medical and social sciences that economic inequality is a major social determinant of health that undermines both health and community resilience (South et al., 2020). This has been brutally exposed during the COVID‐19 pandemic and should raise concerns given the predictions that such widescale disasters are likely to increase over the coming decades as a result of climate change (IPCC, 2021). All three framings of mutual aid analysed above share a sense of “giving” to the vulnerable, who otherwise risk being left behind (Kluge, 2020), whether this is just food, shelter and/or emotional support. But, there is also a sense that some of the mutual aid groups, specifically those in the more radical tradition located in more relatively marginal parts of the world, are offering agency and empowerment to dismantle not only structural inequality, but also all the apparatus that made it possible (Black et al., 2020; Carstensen et al., 2021). To understand this ability to dismantle more specifically, it is vital to think of the various conceptualisations of vulnerability.

The unique event of the COVID‐19 pandemic is providing the context for this rethinking, as vulnerabilities ripple *inwards* from the margins of society to those who found themselves temporarily vulnerable or “newly poor” (World Bank, 2020). So, while we may not truly be “all in *it* together” (as elected officials often proclaimed), numerically more of us are in *it*, and in *it* more deeply than we are used to. In other words, crisis relief mutual aid in the post‐COVID world requires a deeper understanding of the spatial processes of how people become vulnerable (and subsequently resilient and/or resourceful) in the first place.

To be “vulnerable”, however, is a contested and variegated state that is determined by social status, identity, geography and availability of resources (McLaughlin & Dietz, 2008). Significant numbers of people have been made more *financially* vulnerable by the COVID‐19 pandemic due to the increased precarity of their socio‐economic circumstances (Mikolai et al., 2020). For instance, those who do not receive sick pay for time off work (Power et al., 2020), those whose workplace conditions do little to protect them from exposure but who feel unable to complain for fear of dismissal (Apostolidis & McBride, 2020), or who work in sectors where short‐term contracts or casual work are the norm, including gig economies (Mallett, 2020) academia (Kınıkoğlu & Can, 2020) and the arts (Comunian & England, 2020). In many cases, what is being made “vulnerable” is the stretched household or personal finances that provide no resilience against even short‐term shocks (De Ruyter & Hearne, 2020), given that the pandemic happened too quickly for more State‐organised welfare provision to step in immediately.

More generally, the effects of the COVID‐19 pandemic, or more accurately, the inability of some governments to respond quickly or efficiently enough to those most in need, meant that existing social inequalities became exacerbated. For example, in the UK (where the government's pandemic response has been criticised as one of the worst in the developed world; Fletcher et al., 2020), the increase in people struggling to make ends meet during the pandemic led to a 33% increase in those turning to food banks (Trussell Trust, 2021), saw 174,000 renters at risk of eviction or of falling into debt to meet their rent payments (Shelter, 2021), and left 51% of low‐income households unable to afford the IT hardware and WiFi access required for their children's schooling (Hung & Wati, 2020). All this while billionaires in the UK alone increased their wealth by £106.5 billion during the pandemic in 2021 (Chapmen, 2021).

With all this expanding inequality, it is no wonder the explosion in mutual aid efforts moulded around and in response to those at the sharp end, catering to the need born out of the increases in people's *vulnerability*. But within charity versions of “mutual aid” efforts, vulnerability was often perceived only within these financialised contours (Firth, 2020). Those in need were often “identified” first via existing registers of vulnerability, e.g. families already receiving social services from local government, school link workers identifying needy children, people who frequented foodbanks, etc. (Carstensen et al., 2021). “Vulnerability” in this case was a pre‐existing state, one pre‐defined by “official” institutional settings and frameworks. People may have found themselves falling into that category via a drop in their salary, but the “definitions” of vulnerability to these mutual aid groups changed minimally. Hence, charity versions of mutual aid not only ossified existing capitalistic frameworks of philanthropy and volunteerism; they (inadvertently) brought more people into the realm of the vulnerable.

But the term “vulnerability” within geographical literature is more nuanced than this institutional reading affords, and is often embedded within (or indeed superseded by) the musings on resilience, notably in relation to post‐disaster spatialities (see Weichselgartner & Kelman, 2015). As was noted above in the “contributory” model of mutual aid, “resilience from below” can be utilised as a means to counter the State‐led version of “resilience from above” that “in the face of the urgent crises of climate change … actually serves to naturalize the ecologically dominant system of global capitalism” (MacKinnon & Derickson, 2013, p. 266). Such readings of vulnerability (i.e., in association with contributory forms of mutual aid) broaden its conceptualisation out to include the notion of the “community”, be that place‐based (a neighbourhood) or identity‐based (a specific group of raced, ethnic, classed or religious people). But when that community itself has existing systemic vulnerabilities, then contributory models of mutual aid will only bring those groups *back* to a state of pre‐existing vulnerability. For example, Black and Minority Ethnic communities were both more likely to suffer from the underlying health conditions that made people more susceptible to severe symptoms of COVID‐19, because they were more likely to encounter and contract the virus due to over‐representation in the healthcare workforce (Chaudhry et al., 2020), the “grey” economy (Donà, 2021), public‐facing and service jobs (Mamluk & Jones, 2020) and under‐representation in those able to work from home (Kirby, 2020). Their health was made vulnerable by the structural inequalities that have impacted their lives, and which clustered them in low‐income jobs that left them dependent on public transport and jobs involving multiple human interactions. All the while there was a palpable and ugly sense that the media, egged on by government officials looking to shirk responsibility, simultaneously blamed them for moving outside home and between homes, all the while fetishising them as “essential workers” (Cole & Dodds, 2021; Sturm et al., 2021). Hence, their vulnerability was already collective such that it put them at a disproportionate – and fatal – disadvantage when the pandemic struck. In addition, a report by the Institute of Fiscal Studies in June 2020 found that northern and coastal parts of the UK were hit hardest by the pandemic in terms of number of hospitalisations and deaths, a geographical disparity they argue was because of health and income inequalities (Davenport et al., 2020).

So to reconceptualise mutual aid as more mutual, then a more radical version of vulnerability needs to be considered in alignment: one that conceptualises vulnerability not as a bounded state that a person is *subject*ed to, but instead complicates the notion of the vulnerable subject altogether; in other words, viewing vulnerability as a constitutive and emancipatory process, and not a reductive and confining one. Vulnerability is something to embrace because it forces a connection with the Other, and not something to hide (Butler, 2014). Such a view is forwarded geographically by scholars such as Brice (2020), who critiques the traditional “subject” via queer and trans ideologies, arguing that vulnerability becomes a process of creating new collectivities via solidarity, care, empathy and defiance. Indeed, as Butler et al. (2016, p. 1) have suggested, what if “vulnerability were imagined as one of the conditions of the very possibility of resistance”? In these readings, vulnerability is viewed less as “paternalistic” and “understood only as victimisation and passivity” (Butler et al., 2016), but as a site pregnant with new subjectivities of resistance to such paternalistic, victimisation and passivity. Being vulnerable, then, is not to be a passive recipient of aid, someone who needs a bit of free food in order to be an employable subject one again. It is a state of social possibility; of latent connectivity with others to create more just ways of organising communities.

For example, Carstensen et al. (2021) collate research data from parts of the global majority world of how the pandemic brought communities together to create new forms of solidarity. For example, in Kenya, “the spirit of ‘*harambee*’ – that is, people uniting in times of crisis and need – was indeed prominent in Kenyans taking action to support their fellow citizens” (Carstensen et al., 2021, p. 158). They argue that this had longer‐term positive effects of levelling gender pay gaps in communities, boosting youth employment, abolishing interest rates on loans and other progressive benefits (Carstensen et al., 2021). Similarly, when documenting how migrants and refugees in the US were coping with the Trump administration's harsh treatment during the pandemic, Boris (2022, p. 78) found that “despite exclusion from state‐sponsored relief during the pandemic, migrant workers – whether labouring in homes, fields, warehouses, or medical facilities – continued to organise” and as result, they were able to feed, shelter and emotionally support each other, when the US state had abandoned them. Also Vaccarino‐Ruiz et al. (2021, p. 121) show how in Santa Cruz, “social toxicity and possibility [sat] side‐by‐side” in the pandemic, with vulnerable communities using their inability to drive for food (as per State law) to create local food exchanges within walkable neighbourhoods. And, as the results of pandemic‐related research from geographers (and the broader social sciences) begins to be published, there will no doubt be further similar examples.

Vulnerability, as Brice (2020, p. 669) argues “marks the space of possibility for radical social transformation”. It is easy, therefore, to see why this reading of vulnerability is so important for mutual aid. Bringing the radicality of mutual aid together with vulnerability as a site of resistance foregrounds how new spaces of solidarity can be opened up in direct opposition to oppressive frameworks of state‐led articulations of “resilience” (that too often narrate a vulnerable subject as passive, helpless, individualised and “in need” of charity versions of aid). Hence, thinking mutual aid alongside a more constitutive articulation of vulnerability creates a site of possibility for radical new forms of organising society based on solidarity, not charity.

## 4 CONCLUSION: WHERE NOW FOR MUTUAL AID?

The impacts of the COVID‐19 pandemic extend beyond the many tragic deaths and long‐term conditions caused by the virus that a huge number of people have had to endure. It has also impacted household incomes, housing security, labour market engagement and well‐being. Yet, despite the many horrors and insidious consequences of the COVID‐19 pandemic, we have already witnessed signs that there has been a reconfiguration of the way people are cared for and supported in our communities; some even aligning with the more radical anarchist mutual aid characteristics that Kropotkin first espoused. This support has arisen from the pre‐pandemic work of particular individuals, engaged communities and organisations as well as the spontaneous actions of others who mobilised at the start of and during the pandemic. There has been a reawakening of mutualism, in the UK and internationally, at a scale not seen for decades, each enactment itself focused at a local and very intimate level but with the support of other, similar groups who see themselves and their struggles reflected in others (Chevée, 2021; Lachowicz & Donaghey, 2021). At the outset of the pandemic, many forms of community support were classified as “mutual aid” and these covered a diverse range of activities across a spectrum of approaches, enacted across the canvas of local communities. But as this paper has shown, mutual aid has a history, geography and conceptualisation that require a much closer inspection to understand the benefits it offers to the post‐pandemic world.

As its geographical reach tends to be local or even hyper‐local, mutual aid naturally exhibited different characteristics in different locations, and this has ramifications for how mutual aid should be studied in post‐pandemic times. This paper has sought to take the COVID‐19 pandemic and explore how we, as a global community all affected by the same pandemic, mobilised to support and care for one another, and bring this knowledge to bear on the radical geographical literatures of mutual aid. Our analysis leads us to a new conceptual framework of mutual aid that revivifies its radical edge in conjunction with particular readings of vulnerability (e.g., Brice, 2020; Butler et al., 2016) to help us understand how we might draw strength from the emergency we have experienced for the coming catastrophes. In typically prophetic voice, Solnit (2016, p. 13) reminds us that “inside the word emergency is emerge; from an emergency new things come forth. The old certainties are crumbling fast, but danger and possibilities are sisters”. Within the discipline of geography specifically, the conceptual analysis of mutual aid this paper proffers can be transposed, adapted and moulded into important work on intersectional social justice, including (but of course in no way limited to) food insecurity (Williams & May, 2022), squatting and housing rights (Vasudevan, 2015), anti‐raid activism (Elliott‐Cooper, 2021), poverty alleviation (Shaw, 2019), the worker co‐operative movement (Rossi, 2015) and many other ways in which people are actively seeking more democratic ways of organising beyond unjust versions of capitalist realism (Fisher, 2009).

But even beyond the academy, this paper signposts possibilities for an alternative, politically motivated ideology of collective responsibility, co‐operation and mutual survival first envisaged by Kropotkin more than a century ago; one in which the emergence and recovery from the COVID‐19 pandemic takes a community‐focused approach in which vulnerabilities are viewed less as static variables to be countered, but as spaces of radical emancipation from the injustices of capitalist systems that created the vulnerabilities in the first instance. If this is to be pursued, policy actors, local authorities, civil society, academics and activists can build on the good practice that has been enacted and embed it into future resilience planning from the bottom up (South et al., 2020).

The impact of COVID‐19 has been planetary (Mould, 2021) and whilst we are slowly emerging from this particular crisis, we are already caught in the fight for our lives as we slowly come to terms with the climate emergency. The report from the Intergovernmental Panel on Climate Change (Mould, 2021) makes it clear that business as usual is not an option, that new and drastic changes are needed in order to steer ourselves away from planetary destruction for humans and our ways of life. 2021 saw a glut of supposedly “once in a generation” weather events happen consecutively: wildfires in the US and Canada which saw entire towns destroyed; deadly floods in Sierra Leone, China, Germany and Belgium; and Hurricane Ida caused fatalities in New Orleans and New York City. The aftermath of these events are still being played out, but it is already clear that mutual aid groups have mobilised quickly to safeguard those most in need. Sadly, these tragedies mark the beginning of many more extreme weather events that will blight the coming years. But there are lessons we can learn from COVID‐19 that can inform our response to the climate emergencies of the future (Cole & Dodds, 2021), and mutual aid provides a strategy and an ideology for starting to envisage how we might do things differently in the future to avoid such events from becoming catastrophes. This paper aims to contribute to helping the geographical understanding of how we can draw on the strength of humans and our implicit mutualism. By taking stock of our response to the tragedy of COVID‐19, we can perhaps prevent more tragedies from occurring.

## Data Availability

There is no primary data in this article, hence there is none to be made available. The wider project though has all of its outputs and data available online at mutualaid.uk.
